# Biological function of Foot-and-mouth disease virus non-structural proteins and non-coding elements

**DOI:** 10.1186/s12985-016-0561-z

**Published:** 2016-06-22

**Authors:** Yuan Gao, Shi-Qi Sun, Hui-Chen Guo

**Affiliations:** State Key Laboratory of Veterinary Etiological Biology and OIE/National Foot and Mouth Disease Reference Laboratory, Lanzhou Veterinary Research Institute, Chinese Academy of Agricultural Sciences, Lanzhou, Gansu 730046 China

## Abstract

Foot-and-mouth disease virus (FMDV) represses host translation machinery, blocks protein secretion, and cleaves cellular proteins associated with signal transduction and the innate immune response to infection. Non-structural proteins (NSPs) and non-coding elements (NCEs) of FMDV play a critical role in these biological processes. The FMDV virion consists of capsid and nucleic acid. The virus genome is a positive single stranded RNA and encodes a single long open reading frame (ORF) flanked by a long structured 5ʹ-untranslated region (5ʹ-UTR) and a short 3ʹ-UTR. The ORF is translated into a polypeptide chain and processed into four structural proteins (VP1, VP2, VP3, and VP4), 10 NSPs (L^pro^, 2A, 2B, 2C, 3A, 3B_1–3_, 3C^pro^, and 3D^pol^), and some cleavage intermediates. In the past decade, an increasing number of studies have begun to focus on the molecular pathogenesis of FMDV NSPs and NCEs. This review collected recent research progress on the biological functions of these NSPs and NCEs on the replication and host cellular regulation of FMDV to understand the molecular mechanism of host–FMDV interactions and provide perspectives for antiviral strategy and development of novel vaccines.

## Background

Foot-and-mouth disease (FMD), an acute highly contagious viral disease in susceptible cloven-hoofed animals, was described 100 years ago. The etiologic agent, FMD virus (FMDV), is a positive-sense, single-stranded RNA virus that belongs to the *Aphthovirus* genus, *Picornaviridae* family. FMDV is one of the most contagious viruses in cloven-hoofed animals and can cause both acute and prolonged, asymptomatic but persistent infection [1]. Upon infection of susceptible species, FMDV proliferates rapidly and causes vesicular disease in feet and mouth.

The RNA virus genome of FMDV displays a very high mutation rate because the virus-encoded RNA polymerase lacks a proofreading mechanism [2, 3]. The high mutation rate of FMDV, coupled with its rapid proliferation and extensive population, result in the rapid evolution of this virus [4], which contributes to the existence of seven main serotypes (A, O, C, Asia1, South African Territories (SAT) 1, SAT2, and SAT3). In addition, numerous variants and subtypes have been further evolved from each serotype [1]. Given that cross-reactivity varies, antigenic diversity among these serotypes have to be considered during vaccine development [5].

**Fig. 1:**
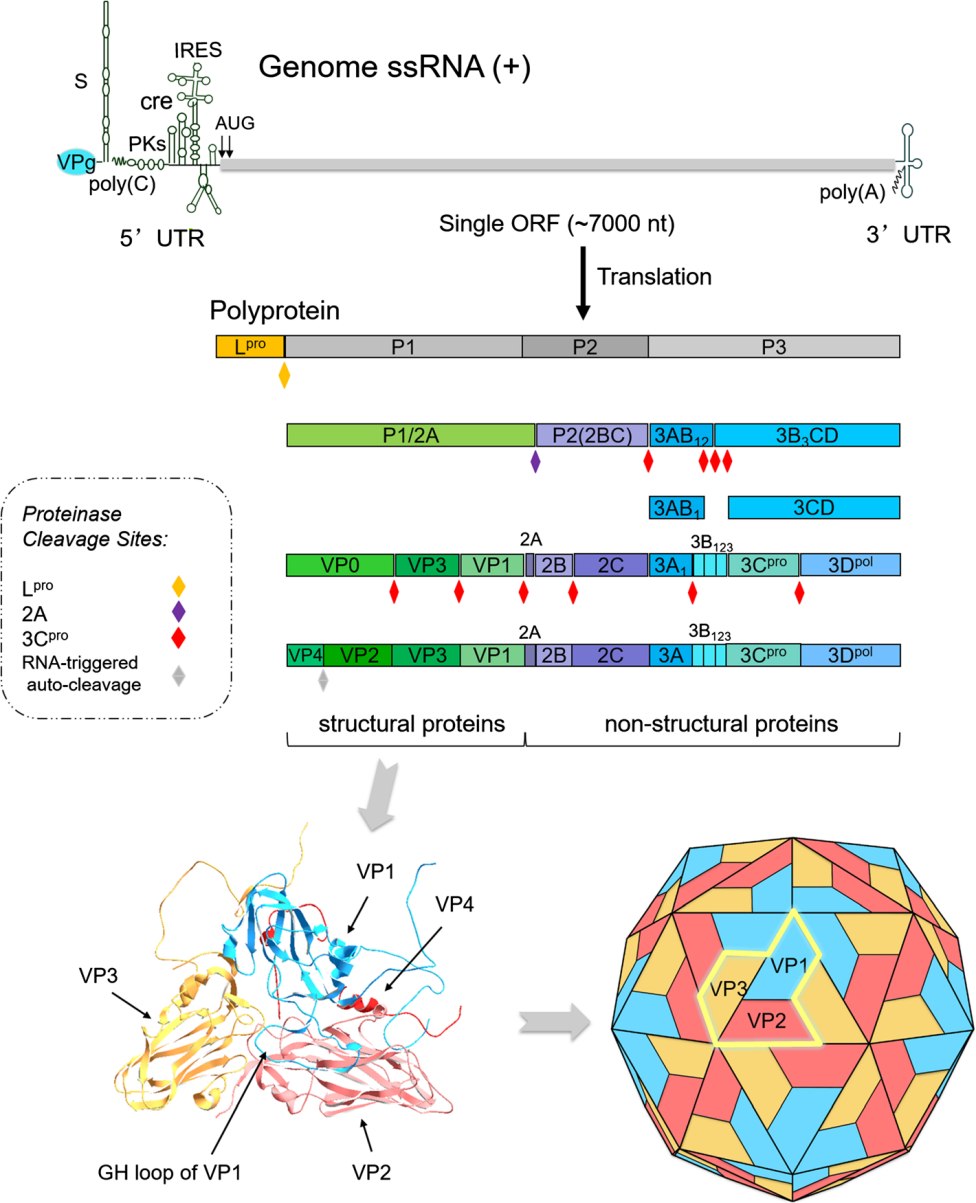
Schematic diagram of FMDV genome, processing of viral polypeptide and conformations of the structural proteins. FMDV genome RNA contains a single open reading frame (ORF) of about 7 kb with two alternative initiation sites. The ORF is flanked by a long 5ʹ-untranslated region (5ʹ-UTR) and a short 3ʹ-UTR. 3B (VPg) is covalently bound to its 5ʹ end. The ORF region is generally divided into four functional areas (L, P1, P2 and P3) due to the different functions of mature polypeptides. ORF-encoded polyprotein is processed into four products, L^pro^, P1-2A, 2BC and P3 by L^pro^, 2A and 3C^pro^. The precursors P1-2A, 2BC and P3 are further processed into mature viral proteins and some cleavage intermediates with relative stability, such as VP0 or 1AB, 3ABC, 3BCD, 3AB, and 3CD by 3C^pro^. Structural proteins form the biological protomer and viral capsid

FMDV virion has a symmetric protein shell (or capsid) enclosing the genomic RNA. Genome RNA contains a positive single-strand chain approximately 8.3 kb long and encodes a single long open reading frame (ORF) of about 7 kb with two alternative initiation sites. The ORF is flanked by a long 5ʹ-untranslated region (5ʹ-UTR) and a short 3ʹ-UTR, and ends with a genetically encoded poly-(A) tail [6]. A genome-linked viral nonstructural protein (NSP), 3B (also known as VPg) containing 23–24 amino acid (aa) residues, is covalently bound to its 5ʹ end, although this protein is rapidly released into an infected cell and is deemed to play no part in translation initiation [7]. The viral ORF can be translated into a polyprotein of about 250 kDa, which is subsequently cleaved by two virus-encoded proteinases (leader (L^pro^) and 3C^pro^) to yield structural and NSPs [8, 9] (Fig. 1).

The FMDV genome was completely sequenced, and all cleavage sites involved in the processing of polypeptides were also identified in the past two decades. Generally, the ORF region in FMDV genome is artificially divided into four functional areas due to the different functions of mature polypeptides [10], which are shown as follows (Fig. 1): L region, which is located at 5ʹ end to the capsid component and codes for L^pro^. P1 region, encoding a precursor for capsid polypeptide, which can generate four mature capsid proteins (VP4, VP2, VP3, and VP1) upon cleavage by viral protease. P2 region encodes three viral proteins (2A, 2B, and 2C) in the middle region of the genome. And P3 region, which encodes four viral proteins: 3A, 3B, 3C^pro^ and 3D^pol^, in which, 3C is a viral protease and 3D an RNA-dependent RNA polymerase [11]. Actually, primary polyprotein is not strictly processed into four products as the functional regions by initial protease, but L^pro^, P1-2A, 2BC and P3 by L^pro^, 2A and 3C^pro^. The precursors P1-2A, 2BC and P3 are further processed into mature viral proteins and some cleavage intermediates with relative stability, such as VP0 or 1AB, 3ABC, 3BCD, 3AB, and 3CD by 3C^pro^ (Fig. 1). Usually, the intermediates may perform functions other than those of their individual constituents.

**Fig. 2:**
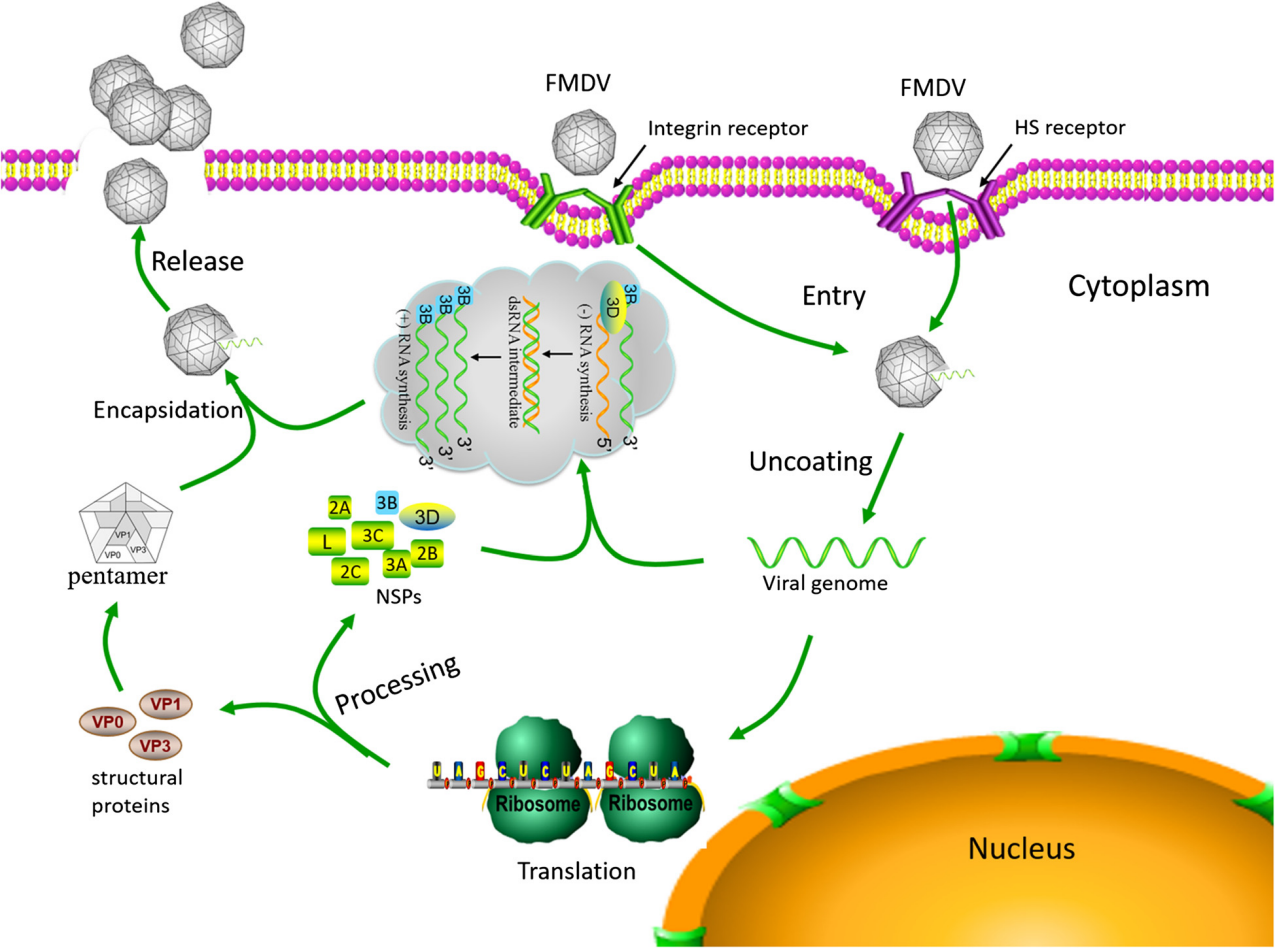
Life cycle of FMDV in host cells. NSPs, non-structural proteins. HS, heparan sulfate. Green line, viral positive-strand (+) RNA. Orange line, viral negative-strand (-) RNA

The virus capsid consists of 60 copies of each of the four structural polypeptides (VP1 to VP4), which are self-assembled into an icosahedral structure with a diameter of 30 nm [12, 13] (Fig. 1). Studies on structural information and protein interaction have shown that the structural protein or the precursor products VP0 (VP2/4 or 1AB), VP1 (1D), and VP3 (1C), which are encoded by P1 region, form immature protomers through weak chemical bond interaction. Then, pentamers are assembled by five protomers [14]. After self-assembly of pentamers to generate an empty capsid, the viral genomic RNA covalently linked to VPg at the 5ʹ end enters the capsid to produce pro-virion. Then the pro-virion is eventually processed into a mature virion following the RNA-triggered auto-cleavage of VP0 [15]. Finally, the virion particles with complete assembly are released from the infected host cells (Fig. 2).

Viral non-coding elements (NCEs) and NSPs play essential roles in FMDV-induced viral evasion. This review focus on the molecular biology of FMDV, along with the functional roles of FMDV NSPs and NCEs in viral replication and virulence, to elucidate how FMDV evades the host immune response and evolves into such an aggressive pathogen.

## Functions of FMDV NCEs

### 5ʹ-UTR

The 5ʹ-UTR is a central element that initiates replication and translation of the picornavirus genome. Similar to other picornaviruses, the FMDV RNA genome does not contain the 5ʹ-terminal cap structure (m7GpppN…), which can be recognized by the translation initiation machinery in all eukaryotic cells [16]. Instead, a short viral protein, 3B or VPg, is covalently linked to the 5ʹ end of the viral RNA [8]. FMDV is unique among the picornaviruses as it encodes three non-identical copies of 3B in tandem (see Section 3.3).

**Fig. 3:**
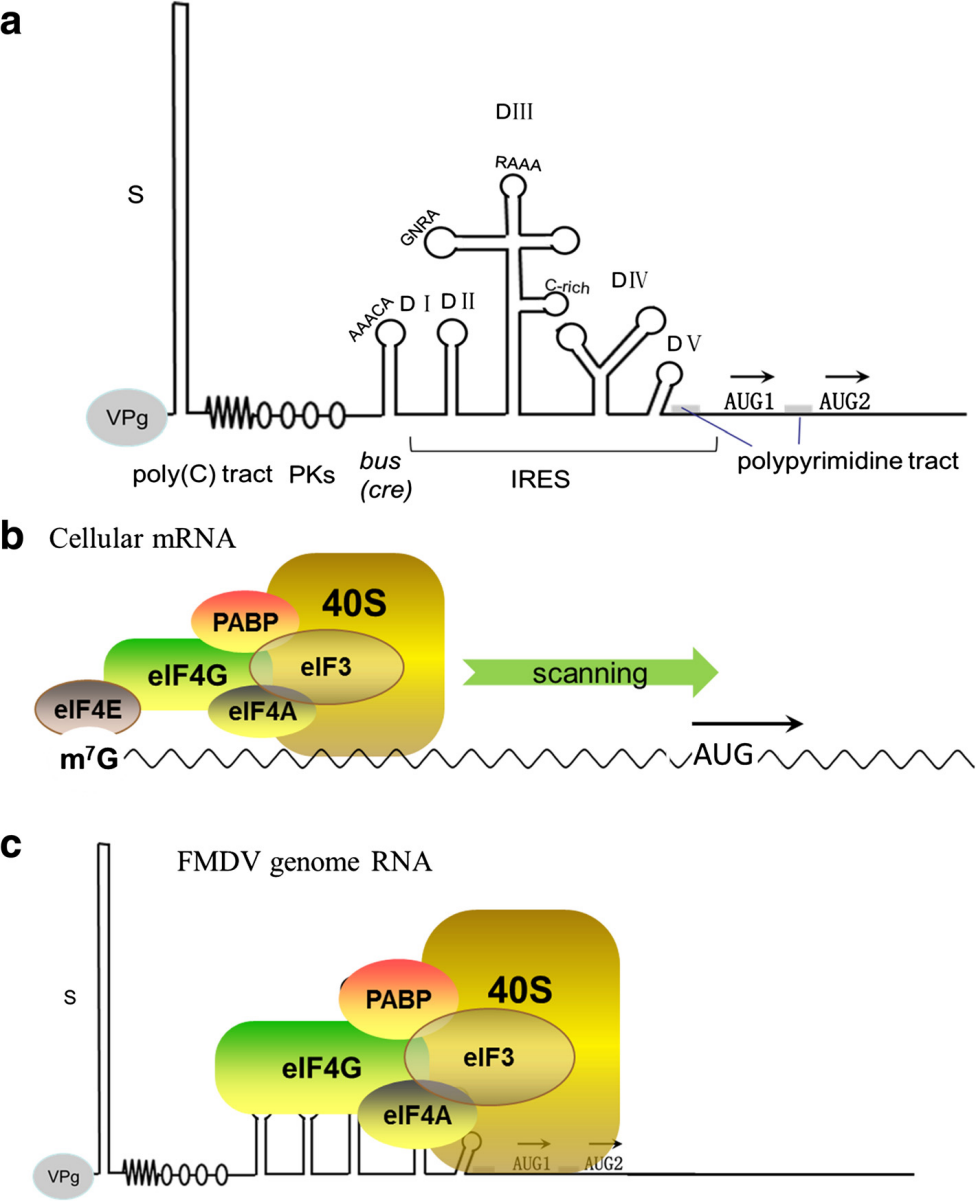
FMDV 5ʹ-UTR provides structural basis for initiation of viral protein translation. **a** Schematic representation of the structural elements within the FMDV 5ʹ-UTR. The 5ʹ-UTR is highly structured and comprises S-fragment, poly(C) tract, 3B-uridylylation site (bus) or cis-acting replication element (cre), pseudoknots (PKs), and internal ribosome entry site (IRES). The organization of the IRES element in different domains (I, II, III, IV, and V) is indicated. Domain I (the first 20 nt of the IRES) belongs to the right arm of bus. The conserved AAACA, GNRA, RAAA motifs, C-rich loop and polypyrimidine tracts are also indicated. The two different initiator codons AUG1 and AUG2 are separated by 84 nt. Note that the stems indicated are not perfectly base-paired. **b** Intact eIF4G is required in cap-dependent protein synthesis of cellular mRNA. The initiation factors are required for the assembly of 48S initiation complex on a capped cellular mRNA. **c** Involvement of translation initiation factors in IRES-dependent protein synthesis. The FMDV IRES elements are indicated. The factors shown are eIF4E (4E), eIF4G (4G), eIF4A (4A), and eIF2 (2) (as part of the ternary complex eIF2/GTP/met-tRNA), along with small ribosomal subunit (40S)

FMDV RNA, which consists of a long 5ʹ-UTR containing over 1300 nucleotides (nt), exhibits extensive secondary structure and is commonly divided into five regions (Fig. 3). The first element of the 5ʹ end is the S-fragment with approximately 350 nt long. Its sequence is highly base-paired and capable of folding into a long stem-loop. Although this highly structured segment has not been extensively studied, this portion is presumed to prevent host exonuclease function to maintain viral genome stability and replication [8].

Following S-fragment is a poly(C) tract exhibiting a variable length of 150–200 nt in typical field strains. Investigations on genetically engineered viral genome revealed that a certain threshold length of poly(C) is required to rescue live virus, although no evidence has shown that poly(C) tract length is directly associated with virulence [17]. On the 3ʹ end of the poly(C) tract is a variable region folded as multiple pseudoknots (PKs) (2–4) [18, 19]. The function of this element remains unknown, but it is possibly associated with the poly(C) tract [16].

Downstream of the PK region lies a 3B-uridylylation site (*bus*), also known as *cis*-acting replication element (*cre*), which is a highly conserved stem-loop of 55 nt and is essential for viral genome RNA replication [20]. This element is commonly consisted in different picornavirus RNAs, and all picornavirus *bus* elements contain a conserved “AAACA” motif in the loop region. This motif acts as the template for uridylylation of VPg (3B) to produce VPgpU and/or VPgpUpU, collectively termed as VPgpU(pU), by the viral RNA polymerase 3D [21, 22]. VPgpU(pU) serves as a primer that initiates viral RNA synthesis. Interestingly, most known viral *bus* structures are embedded in protein-coding regions of the viral genome, except for that of FMDV, which has a *bus* structure that lies in the 5ʹ-UTR region [8]. Alteration in the consensus “AAACA” motif disrupts viral genome replication but does not significantly affect RNA translation in FMDV [20]. Moreover, FMDV is temperature sensitive (ts), and viral replication is greatly suppressed at non-permissive temperature. Mutant analysis reveals that the ts element is located within the *bus* sequence [23], indicating that “*cre*” also functions in *trans*.

The last portion of the 5ʹ-UTR is the internal ribosome entry site (IRES) element, a type II IRES element [24]. Consisting of approximately 450 nt, the IRES element is required for cap-independent internal translation initiation of viral RNA [20, 25]. FMDV IRES has five domains and forms multiple stem-loops (Fig. 3a). There are evidences to support that these domains are involved in translational control. Some highly conserved sequences exist in these domains, like Domain II containing a polypyrimidine tract (UCUUU) that provides a polypyrimidine tract-binding protein (PTB) binding site [26]. Domain III includes two conserved essential motifs, GNRA and RAAA (N is any nucleotide and R stands for purine) at the apical region, and a conserved C-rich loop at the middle region [27, 28]. Modification of the 5ʹ-G or 3ʹ-A residue of GNRA both greatly diminish the activity of FMDV IRES [27, 29] and mutation of RAAA shows abolished activity of FMDV IRES [28, 30], suggesting that they are important for IRES activity. This domain also plays a key role in RNA–RNA interactions and RNA–protein interactions in FMDV [31]. Domain IV is arranged into two stem-loop structures containing A-rich internal bulges in FMDV, and it is responsible for the interaction with eIF4G, an essential translation initiation factor for IRES-mediated translation initiation in FMDV [32, 33]. Domain V consists of a conserved hairpin-loop region and a polypyrimidine-rich tract sequence at 20 nt upstream of the initiation codon AUG [34], which is crucial to identify and initiate viral protein synthesis. Some mutations in this region are highly detrimental to IRES activity in FMDV [25] (Fig. 3a).

### Translation of FMDV RNA

The featured IRES element is an essential structural region for initiation of protein synthesis in picornavirus genome RNA [25, 35, 36]. The IRES is a *cis*-acting RNA sequence that adopts diverse three-dimensional structures to recruit the translation machinery using a mechanism that is independent on the 5′end [37]. Nearly all canonical cellular translation initiation factors are required for IRES-dependent initiation of translation in FMDV except eIF4E, a cap-binding protein [16, 38], consistent with the fact that FMDV genome RNA lacks the cap structure on the 5ʹ end (Fig. 3b and c). For most cellular mRNAs, the 5′cap structure is recognized by eIF4F, a trimeric complex composed of eIF4A (an RNA helicase), eIF4E (a specific cap structure-binding protein), and eIF4G (a scaffold protein). The scaffold protein eIF4G interacts with eIF4A, PABP and the multimeric factor eIF3. The complex bound to the 40S ribosomal subunit is recruited to the mRNA along with other factors, resulting in eukaryotic protein synthesis [38] (Fig. 3b). Cleavage of eIF4G by viral L^pro^ removes its N-terminal portion, the binding sites for eIF4E, impairing cap-dependent protein synthesis in host cells. By contrast, the C-terminal portion of eIF4G retains the binding sites for eIF4A and eIF3, which is sufficient for FMDV IRES activity.

However, viral IRES are characterized by the presence of ignored AUGs upstream of the functional start codon, heavy RNA structure and high GC content [39, 40]. Despite being unable to direct cap-dependent translation, the C-terminal portion of eIF4G is fully efficient in FMDV IRES-driven translation [16, 41]. Reconstitution assays have demonstrated that assembly of initiation complexes into IRES element requires either the intact type or C-terminal fragment of eIF4G, in addition to eIF4A, and eIF3 [42, 43]. Moreover, eIF4G, either the intact type or the C-terminal cleavage product, directly interacts with IRES in domains III and IV [44]. eIF4A and eIF3, which bind to eIF4G, also indirectly interact with IRES to participate in IRES-directed translation initiation. eIF4B, another factor that directly interacts with IRES at domain IV, also affects the activity of IRES, although the effect is rather modest [45].

In addition to the eIFs described above, many other cellular proteins are also involved in the modulation (stimulate or repress) of IRES activity. They are all termed IRES-transacting factors (ITAFs), including PTB, PCBP2, the SR splicing factor (SRp20), the far upstream element binding protein 2 (FBP2), the lupus antigen (La), unr (upstream of N-ras), nucleolin, or Gemin5, etc [46].

The polypyrimidine PTB was the first protein identified as an ITAF [25, 47, 48]. PTB contains four RNA recognition motifs (RRM) to recognize U/C-rich sequences. PTB directly binds to polypyrimidine tracts on IRES element to stimulate IRES activity in FMDV [47]. The poly(C) binding proteins, PCBP1 and PCBP2, also recognize and interact with FMDV IRES domain II, but the depletion test showed that such an interaction site is not indispensable for FMDV IRES activity [41, 49]. Moreover, secondary protein–protein or RNA-protein bridges could facilitate IRES activity. As examples, SRp20 enhances IRES-mediated translation via its interaction with PCBP2 [50]. ITAF45 (also known as Ebp1), together with PTB, sharing the same binding region in IRES, cooperatively stimulate FMDV IRES activity [51]. Later studies found some ITAFs of IRES downregulators. For example, Gemin5, a cytoplasmic protein that binds directly to the FMDV IRES and down-regulates translation [52]. Besides, FBP2 negatively regulates EV71 IRES activity [53], and the double stranded RNA-binding protein DRBP76:NF45, is a nuclear heterodimeric protein that interacts with HRV IRES and represses its activity [54].

As a consequence of in depth RNA–protein interaction studies performed with picornavirus IRES, the list of ITAFs is still growing incessantly [55]. The Glycil tRNA synthetase (GARS) emerges as a class of novel ITAFs stimulating picornavirus IRES activity [56]. Predictably, more ITAFs will be identified in the near future, which will help to provide more details about interactions of IRES-proteins within host cells.

### 3ʹ-UTR

FMDV RNA 3ʹ-UTR consists of two components, a structural sequence of 90 nt folding into two separate stem-loops and a poly(A) tail with variable length [16, 57] (Fig. 1). These elements are both involved in viral replication and virulence [24]. Molecular biology studies have demonstrated that the structured 3ʹ-UTR directly binds to S-fragment and IRES elements at two distinct positions in the 5ʹ-UTR through specific long-range RNA–RNA contact pattern [57]. IRES-3ʹ-UTR interaction requires both stem–loop structures of 3ʹ-UTR, which stimulates IRES activity and is independent of the poly(A) tail. Whereas the S-fragment interacts with each of the stem–loops and is dependent on the poly(A) tail [57]. These findings indicated that the 3ʹ-UTR enhances IRES activity and determines the virulence of FMDV. Moreover, genetic evidence reveals that recombinant FMDV with a deleted structural sequence in 3ʹ-UTR cannot be recovered [58], demonstrating that the structured region in the 3ʹ-UTR is essential for FMDV infectivity and replication.

In addition to direct RNA-RNA interaction, 5′–3′ end bridges could also involve protein-protein and protein–RNA interaction. Studies found that cellular proteins PCBPs and p47 can both interact with the S region and 3ʹ-UTR by directly binding to them [57]. As mentioned in the above section, SRp20 directly interacts with PCBP2, and Ebp1 cooperates with PTB to stimulate viral genome translation, raising the possibility that secondary protein–protein bridges take important roles in RNA–RNA interaction between 5ʹ and 3ʹ ends to modulate the viral genome translation in FMDV.

## Functions of FMDV NSPs

### L^pro^

The viral protein L^pro^ is a region in the polyprotein preceding the capsid precursor protein [59]. L^pro^, the first protein in FMDV to be translated, is initiated at two different start codon AUGs separated by 84 nt. L^pro^ has two alternative forms, namely, Lab^pro^ and Lb^pro^. Both forms have been detected *in vitro* and *in vivo* [60, 61]. Lb^pro^ protein (synthesized from the second AUG) is the major protein type *in vivo*. Viable viruses can be recovered from synthetic genomes containing mutations in the first AUG but not in the second [62, 63]. The sequence between the two AUGs are possibly involved in start codon recognition through interactions with a regulatory factor [62, 64]. FMDV L^pro^ is a well-characterized papain-like proteinase [65–67] that releases itself from the polyprotein via cleavage between its own C-terminus and the N-terminus of VP4 at the sequence ArgLysLeuLys ↓ GlyAlaGlySer during viral maturation [66, 68].

L^pro^ is an important determinant of virulence. Previous studies showed that the L^pro^-deleted virus shows only a slightly slower replication rate than the wild-type (WT) virus [63] but exerts a dramatically lower ability to cause lesions during intradermal injection [69] and fails to cause clinical signs of FMD when exposed to aerosol containing high doses of leaderless virus in cattle and swine [70, 71]. Thus, L^pro^ is not required for viral replication but is indispensable for the pathogenesis of FMDV.

L^pro^ also represses host cell translation by cleaving the translation initiation factor eIF4G [8, 66]. Strong evidence confirmed that L^pro^ recognizes and cleaves the crucial host translation initiation factor eIF4G at the site between Gly_479_ and Arg_480_ [66, 72, 73]. eIF4G is a key scaffold protein for the attachment of other translation initiation factors to exert their functions. Cleavage of eIF4G directly shuts off host cap-dependent mRNA translation [73, 74]. By contrast, FMDV RNA, which initiates translation in a cap-independent manner via its IRES element, does not require intact eIF4G. Removal of the N-terminus of eIF4G by L^pro^ does not impair viral RNA translation initiation. Thus, FMDV freely uses the host protein synthesis machinery to synthesize viral protein (Fig. 3) [8].

L^pro^ blocks interferon (IFN) activity directly and indirectly by inhibiting the central upstream regulatory factor [75–77]. IFN induction is one of the most important host innate immune response to viral infection [78]. Secreted IFN proteins bind to neighbor cells by paracrine manner to induce the expression of a number of IFN-stimulated genes to mediate various biological responses [79], including inhibition of viral replication. Notably, infection with leaderless virus, which lacks the L^pro^ coding region, induces higher levels of type I IFN (IFN-α and IFN-β) mRNA level than WT virus, and type I IFN downstream signaling can only be detected in cultures with the leaderless virus infection [75, 76, 80]. Therefore, type I IFNs, including their mRNAs and proteins, are limited by viral L^pro^. L^pro^ also inhibits dsRNA-induced IFN-λ1 expression, which is a type III IFN demonstrating significant antiviral activity against FMDV [77].

No evidence demonstrated the direct cleavage of IFNs by L^pro^. Translational repression of IFNs is probably resulted from the blocking of FMDV L^pro^-induced cap-dependent mRNA translation by L^pro^-mediated cleavage of eIF4G [8, 81]. Further studies have attempted to investigate the molecular mechanism of L^pro^ virulence. Promoter activity and protein studies have shown that FMDV L^pro^ down-regulates interferon regulatory factor 3/7 (IRF-3/7) expression both at the transcription and translation levels [82, 83]. IRF-3/7 are important regulators in RIG-I/MDA5-induced innate immune signaling, a crucial pathway response to infections caused by picornaviruses [84]. Their inhibition suppresses the expression of type I IFNs and downstream cytokines, including IFN-α/β and CCL5, also known as RANTES [82, 83].

A recent study found that FMDV Lb^pro^ is a novel viral deubiquitylation (DUB) enzyme [85]. Ubiquitination and deubiquitination, which are a class of important regulation patterns, are critically involved in many signaling cascades, including virus-induced type I IFN signaling [86]. Viruses are connected to ubiquitin and ubiquitin-like modifiers in a variety of ways [87, 88]. Sequence analyses revealed that catalytic residues (Cys51 and His148) are highly conserved in Lb^pro^, the shorter form of L^pro^, in all seven serotypes. The topology of FMDV Lb^pro^ is remarkably similar to that of some known DUBs, such as ubiquitin-specific protease 14, a cellular DUB [89], as well as severe acute respiratory syndrome coronavirus (SARS-CoV) papain-like protease (PL^pro^), a coronaviral DUB [90]. In addition, Biochemical and molecular evidence also revealed that FMDV Lb^pro^ can remove ubiquitin (Ub) moieties from cellular substrates, function on both lysine-48- and lysine-63-linked polyubiquitin chains, a feature shared by other known viral DUBs, such as human cytomegalovirus UL48, herpes simplex virus type 1 UL36, and SARS-CoV PL^pro^ [91, 92], indicating that FMDV Lb^pro^ exhibits deubiquitinating activity. A new mechanism of L^pro^ blocking IFN antiviral response is revealed by the evidence that L^pro^ antagonizes type I IFN induction by deubiquitinating the critical signaling components RIG-I, TBK1, TRAF3, and TRAF6 [85].

**Fig. 4:**
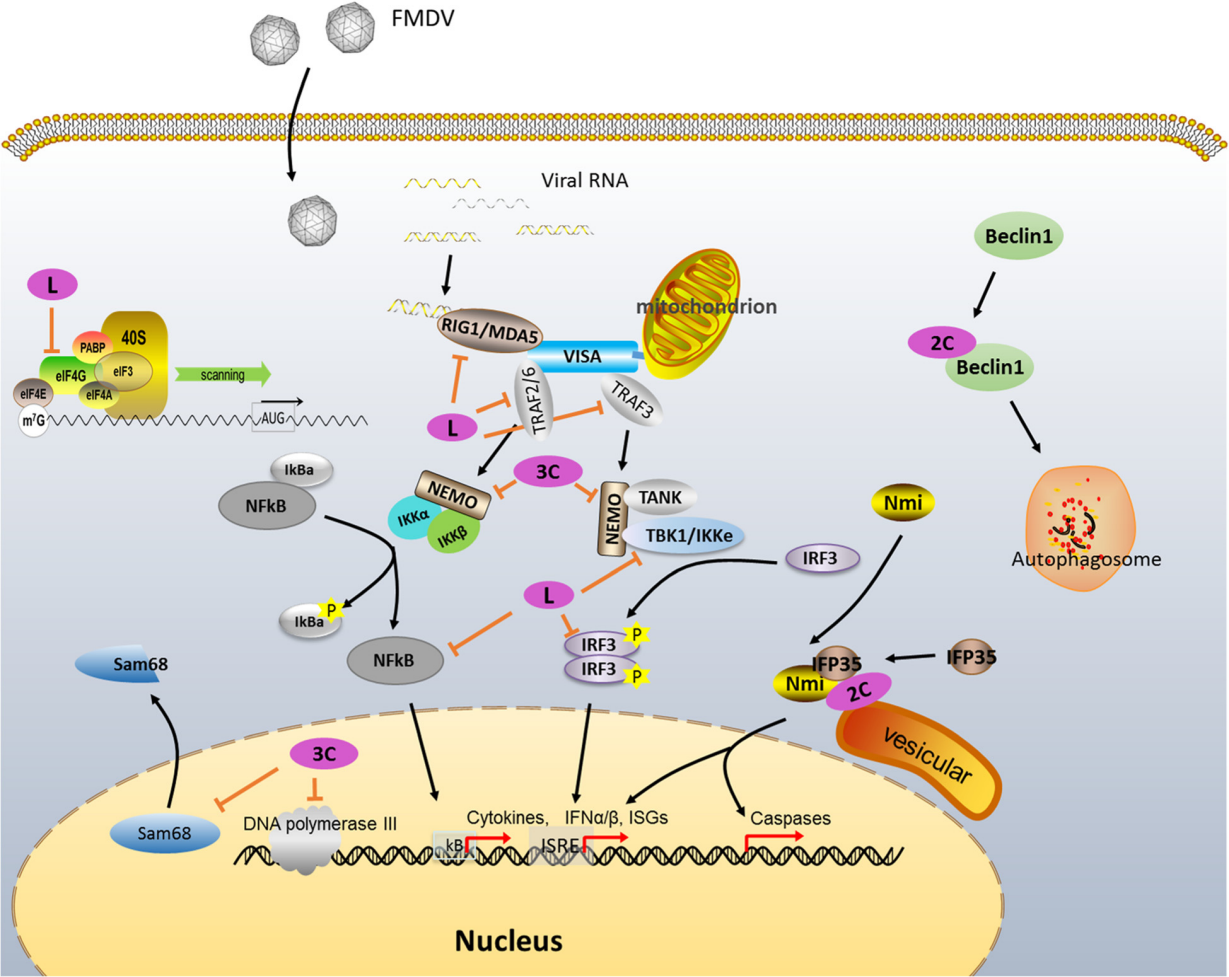
Known functions of FMDV nonstructural proteins on cellular regulation. Purple circles: FMDV nonstructural proteins. Red arrow: activation of downstream gene expression. Orange line with vertical stub: inhibition of downstream proteins. kB, a canonical kB element response to Nuclear factor kB (NF-kB). ISRE, IFN-sensitive response element. Caspases, represent a class of genes inducing apoptosis and inflammation

To sum up, FMDV L^pro^ generally functions as a multifunctional protein that blocks IFN-mediated antiviral response via multiple distinct mechanisms (Fig. 4): (1) L^pro^ shuts off host cell translation through cleavage of the translation initiation factor eIF4G, suppressing IFN protein expression [93]; (2) L^pro^ represses IFN activity by inhibiting activation of central upstream regulatory factors, including NFkB and IRF-3/7 [82]; and (3) L^pro^ acts as a DUB and cleaves ubiquitin chains from RIG-I, TBK1, TRAF3, and TRAF6, thereby inhibiting type I IFN signaling [85].

### 2A Protein

The P2 portion in the picornavirus genome encodes three mature viral proteins, namely 2A, 2B, and 2C (Fig. 1). FMDV 2B and 2C are partially homologous to other picornavirus, whereas FMDV 2A is only an 18 aa peptide [94] and is much shorter than the other picornavirus members but highly conserved with cardiovirus at the 2A/2B junction. The FMDV 2A protein lacks any protease motifs and only contains the characteristic C-terminal motif “-Glu(x)AsnProGly(2A)/Pro(2B)-” [95]. In addition, the conserved cleavage site is located between 2A and 2B Gly(2A)/Pro(2B) [94, 96]. Mutation research confirmed that Gly (2A) is the most important amino acid for cleavage activity at the 2A/2B junction [97, 98], whereas recombinant FMDV sequence containing mutation in the 2A peptide can produce uncleaved proteins. Moreover, cleavage between 2A and 2B only occurs as a co-translational event. Thus, the 2A cleavage event occurs only during polypeptide synthesis [95], such that the 2A peptide remains connected to the P1 structural protein precursor (P1-2A) following primary cleavage of the polyprotein [99]. 2A is cleaved from the P1-2A precursor either by 3C^pro^ or 3CD^pro^ [100].

The FMDV 2A peptide, along with the first aa of the 2B protein, can mediate cleavage in artificial polyprotein systems [94, 95]. However, this FMDV 2A-mediated artificial polyprotein cleavage does not work in the prokaryotic system [94]. In addition, the synthesized upstream proteins of the 2A sequence are always present in greater molar excess than the downstream proteins of the 2A sequence [95]. Thus, the 2A-2B cleavage event is not a proteolytic event but a modification of the translational machinery by the 2A peptide, allowing the release of the protein-2A from the ribosome while permitting the synthesis of the downstream proteins to proceed [95, 96].

### 2B Protein

Picornaviruses 2B are viroporins, a class of low-molecular-weight hydrophobic transmembrane proteins encoded by a wide range of animal viruses. The transmembrane hydrophobic domains interact with the phospholipid bilayer to induce dispersion, increasing membrane permeability and promoting the release of viral particles. Thus, viroporins are crucial for viral pathogenicity [101, 102].

Knowledge on FMDV 2B is limited. FMDV 2B encodes a 154 aa peptide, which is slightly longer than other viroporins and contains two predicted putative transmembrane domains located in 83–104 aa and 119–137 aa, respectively [103]. Topology studies have shown that FMDV 2B is located in the endoplasmic reticulum (ER) and exhibits a transmembrane topology similar to that of class IIB viroporins, which consist of two transmembrane domains and their N- and C-termini both extend to the cytoplasmic matrix [103]. Similar to other known viroporins, FMDV 2B induces damage to the integrity of the host cell’s membrane and causes Ca^2+^ abnormalities, activating autophagy [103]. However, this phenomenon has not yet been fully confirmed in FMDV.

Some reports have indicated that 2B functions synergistically with other viral proteins, such as 2C, in FMDV [104]. Previous findings have also confirmed that picornaviral infections block protein secretion in host cells. Rather than other picornaviruses, ER-to-Golgi apparatus protein transport in FMDV is blocked by the 2BC precursor protein but not by 2B or 2C. Microscopy and immunoprecipitation indicated that the 2BC protein blocks cellular protein transport between the ER and Golgi apparatus during FMDV infection [105], and a study showed that protein transport was abolished only in the case of both 2B and 2C co-expression [104]. This phenomenon is possibly caused by the synergistic effect of 2B and 2C.

Viroporins participate in multiple stages of the viral life cycle, such as cell entry and genome replication. Viruses with viroporin deletion cannot properly assemble and release from cells [102, 106]. Thus, viroporins are attractive targets for antiviral therapy based on recent findings on their structure and biological functions. Several viroporin inhibitors have been developed to effectively suppress viral replication through inhibition of membrane permeabilizing activities. These inhibitors include amantadine, which is used against hepatitis C virus (HCV) p7 and influenza A virus M2 protein; 4,4ʹ-diisothiocyano-2,2ʹ-stilbenedisulphonic acid [107], which is against enterovirus 71 P2B protein; 5-(N,N-hexamethylene) amiloride (HMA), against HIV-1 Vpu, HCV p7, and SARS-CoV E protein; and N-(5-(1-methyl-1H-pyrazol-4-yl)-napthalene-2-carbonyl)-guanidine (BIT225), which can also inhibit HIV-1 Vpu and HCV p7 [102, 108]. Although no reports demonstrated the effect of these drugs on FMDV replication, these findings provide potential strategies for developing antiviral drugs against FMDV viroporin 2B.

### 2C Protein

Protein 2C is one of the highly conserved molecules among the viral proteins encoded by FMDV, and 2C-like (2CL) proteins generally exist in many other RNA viruses across animals and plants [109]. The FMDV protein 2C is a 318 aa polypeptide that contains a predicted amphipathic helix in its N-terminus (residues 17–34) [110, 111]. It is responsible for many biological functions linked to membrane targeting. Although the reports about FMDV 2C are limited now, a number of studies have uncovered the function of other picornaviruses 2C. FMDV 2C is the largest membrane-binding component of the virus RNA replication complex and is speculated to perform a function similar to the 2C in other picornaviruses [104, 112]. 2C plays a key role in both membrane rearrangement and formation of the viral replication complex. In addition, 2C is implicated in virus-induced cytopathic effects [113–116]. Immunofluorescence studies demonstrated that protein 2C aggregates at the cellular periphery in FMDV-infected cells [117] and is inclined to bind to ER [118]. These findings are consistent with those of subcellular fractionation studies, which revealed that 2C co-localizes with the ER membrane, Golgi apparatus, and lysosomes in poliovirus [119–122]. 2C also co-localizes with the membrane-bound replication complex, suggesting that 2C acts as an important factor in viral replication. This function is also confirmed by the inhibition experiments. Guanidine hydrochloride, an antiviral compound and a molecular antagonist against protein 2C can inhibit viral RNA synthesis in picornavirus-infected cells, and virus strains containing guanidine-resistant (gr) 2C mutation cannot be inhibited by guanidine hydrochloride, providing direct evidence that FMDV 2C protein plays a key role in virus replication [123, 124]. This phenomenon is also observed in poliovirus [111, 125, 126]. Notably, FMDV 2C is only present when the replication complex forms during virus proliferation but absent from clarified virus stocks used for vaccine preparation. Thus, 2C protein can be used to differentiate potential carrier convalescent animals from vaccinated livestock [127–130].

Many other functions of 2C in other picornavirus members have been reported, including viral RNA binding activity [131, 132], NTP binding activity [109], ATPase and GTPase activities [133, 134], binding to Reticulon 3, as well as viral replication [135]. Given that 2C proteins are highly conserved among picornavirus members, FMDV 2C is speculated to demonstrate most of these activities. Indeed, some functions of FMDV 2C have been confirmed [136].

A recent report discovered that FMDV 2C is involved in apoptosis induction and type 1 IFN response. Yeast two-hybrid system and immunoprecipitation approaches revealed that FMDV 2C interacts with N-myc and STAT interacting (Nmi) protein [137], a factor involved in multiple cell signaling by interacting with many proteins, including IFN signaling and apoptosis signaling [138–140], as well as its heterodimeric complex partner, IFN-induced 35-kDa protein (IFP35) [137], a protein with potential roles in apoptosis, cytokine response, and antiviral activity [141–143]. Moreover, immunoprecipitation and immunofluorescence studies indicated that 2C can recruit Nmi and IFP35 into the intracellular membrane structure by changing their subcellular distribution, forming the 2C-Nmi-IFP35 complex. Thus, 2C likely induces apoptosis through the host protein Nmi, as well as induces a type I IFN response through the host protein IFP35 [118, 137, 144]. Nevertheless, some studies indicated that apoptosis does not occur in FMDV-infected cells [145, 146]. Further investigation is needed to clarify the underlying mechanism of FMDV and apoptosis.

Some other research reported that FMDV 2C is involved in FMDV-induced autophagy. FMDV triggers cellular autophagy and enhances viral replication [147]. Gladue *et al*. further investigated the interaction between FMDV 2C and cellular Beclin1 using a yeast two-hybrid model, immunoprecipitation and confocal microscopy. They found that FMDV 2C binds to Beclin1, a factor that plays dual roles in the autophagy pathway. Beclin1 is involved in initiation of autophagosome formation and fusion of autophagosome to lysosome [148, 149]. So 2C-induced Beclin1 inactivation blocks the fusion of FMDV-containing autophagosomes to lysosomes and prevents virus degradation [150]. 2C-Beclin1 interaction plays a significant role in virus replication [150].

Viral 2C protein is speculated to act as an important regulator integrating multiple cell signaling during FMDV infection, including apoptosis, immune response, and autophagy. This protein reduces the cellular killing effect against viruses and promotes virus survival and proliferation, thereby facilitating viral proliferation and release of virus particles (Fig. 4).

### 3A Protein

FMDV 3A protein is a 153 aa peptide, which is larger than other picornaviral 3A protein, like the 87 aa-long poliovirus 3A [8]. FMDV 3A is conserved in most FMDV strains. Half of the 3A coding region in the N-terminus (positions 1–75) encoding a hydrophilic domain and a hydrophobic domain capable of binding to membranes, is highly conserved in all FMDV strains [151]. By contrast, many mutations and deletions occur in the C-terminus of all FMDV strains.

Based on its hydrophobic motifs, FMDV 3A exerts membrane binding activity [105]. Fluorescent staining revealed that 3A in infected cells partially co-localizes with the ER marker calreticulin and with Golgi stacks protein p58 [152]. In contrast to other picornaviruses, transient expression of 3AB proteins does not induce major rearrangements of intracellular membranes as inferred from immunofluorescence and electron microscopy studies [152]. FMDV 3A preferentially localizes in small vesicles when transiently expressed [105]. Rosas *et al*. [153] generated BHK21 cell lines stably expressing 3A and its precursor 3AB and found that expression levels of 3A and its processors exert varying degrees of cell toxicity but do not induce cell membrane rearrangements [153]. In addition, stable expression of 3A or 3AB protein enhances FMDV replication, including increase of virus plaque formation and virus titers. But transiently expressing 3AB protein shows a decreased level of FMDV infection. These indicated a transacting role of 3A and 3AB on the FMDV multiplication cycle [153].

Reports demonstrated that 3A plays a role in virulence and host range. Some amino acids substitutions or deletions in 3A protein are associated with change in host range and tropism of FMDV [154, 155] and other picornaviruses, including poliovirus and human rhinovirus [156]. In the N-terminus of 3A, the amino acid substitution Q44 to R is sufficient to confer FMDV C-S8c1 strain adaptation to guinea pig [155]. Two kinds of natural deletion mutants were reported in the C- terminus: 10 aa deletion at positions 93–102 and 11 aa deletion at positions 133–143 [154, 157]. The 133–143 deletion in 3A was observed in both cattle and pig isolates and does not affect the host range and virulence of FMDV [151]. By contrast, the 93–102 deletion in 3A is associated with high virulence in swine and is observed in a variant of FMDV serotype O isolated in Taiwan in 1997 (O/TAW/97). This deletion severely affected swine but did not spread to cattle [154, 157, 158], and also reduced virus replication efficiency in bovine cells but not in swine cells [159]. Similar deletion mutants containing 19 aa (O1C-O/E) to 20 aa (C3R-O/E) deletions in 3A protein were also observed in egg-adapted attenuated strains. These mutants displayed reduced virulence in cattle and were used for early vaccine development [154, 158]. Another artificial mutant containing 20 aa deletion at positions 87–106 of 3A demonstrated a significantly reduced replication ability and attenuated virulence in cattle [157]. Although its underlying mechanisms remain unclear, FMDV 3A is another viral protein affecting FMDV virulence, and some positions in 3A are associated with alterations in viral virulence and host range as indicated by the aforementioned studies.

In addition to 2C protein, 3ABC, the 3A precursor, is also used to differentiate potential carrier from vaccinated animals both in cattle and swine [160]. Actually, 3ABC is the most antigenic protein among all NSPs and is the best serological indicator of infection with FMDV [160]. Various ELISAs based on 3ABC antigen or antibody have been developed for discrimination between infected and non-infected animals regardless of their vaccination status [160–163].

### 3B Protein

Protein 3B, which is also known as VPg, is covalently bound to the 5ʹ terminus of the genome and antigenome and primes picornavirus RNA synthesis [8]. In contrast to other picornaviruse that encode a single copy of 3B, the FMDV 3B protein is unique because it exists in three similar but non-identical copies (3B_1_, 3B_2_, and 3B_3_), which are 23–24 aa long [164]. No natural FMDV strains have been reported to contain fewer than three copies of 3B [165], although not all three copies of FMDV 3B are needed to maintain infectivity [164], suggesting that there is a strong selective pressure towards maintaining this redundancy.

Uridylylation of the VPg peptide primer is the first stage in the replication of the picornavirus genome (Fig. 2). The picornavirus genome has a 5ʹ terminal feature of VPgpU(pU) covalently linked, which contributes to the use of VPg as a peptide primer to synthesize viral RNA. This peptide attaches to RNA via a conserved Tyr3 residue through the action of viral RNA polymerase (3D^pol^). In this process, the viral 3D^pol^ catalyzes the binding of two uridine monophosphate (UMP) molecules to the hydroxyl group of this Tyr3 using as template a *cis*-replicating element (*bus*/*cre*) in FMDV genome [166]. Three isoforms of FMDV 3B (3B_1_, 3B_2_, and 3B_3_) can all be uridylylated *in vitro*, although 3B_3_ is likely the most efficient substrate for 3D^pol^ activity [167]. VPg peptide primer uridylylation can be performed *in vitro* using purified components, including VPg (3B) with 3D^pol^, 3CD precursor, UTP, and an RNA template containing a stem-loop structure (*bus*) [167]. VPgpU(pU) are produced during this reaction and synthesis of positive- and negative-sense RNAs is initiated [166, 168].

A reverse genetics study demonstrated that deletion of the 3B_3_ coding sequence exerts a deleterious effect on FMDV RNA replication, resulting in production of a non-infectious RNA transcript [167, 169]. Laboratory recombinant virus lacking 3B_1_ and 3B_2_ also reduces viral RNA synthesis levels and infective particle formation *in vitro*, attenuats disease in pigs, but not drastically [170]. These studies indicated that 3B_3_ is more important than 3B_1_ and 3B_2_ to maintain viral RNA replication, but co-existence of all three 3B copies exerts the best RNA replication efficiency. Whereas the underlying mechanisms of their respective roles on viral RNA replication and how the three copies are integrated during viral replication still require further investigation.

### 3C^pro^

FMDV 3C proteinase, responsible for most cleavages of viral polyprotein, was recently identified as a chymotrypsin-like cysteine protease [8, 171], although its function and catalytic residues were reported since 1995 [172]. Crystal structure and mutagenic research in FMDV demonstrated that a featured apolar surface loop containing a β-ribbon structure that folds over peptide binding cleft and clearly contributes to substrate recognition is important for catalytic activity. In addition, Cys142 in the Cys-His-Asp/Glu catalytic triad at the tip of the β-ribbon significantly affects enzyme activity [173, 174]. Except for the autocatalytic cleavage of L^pro^ from P1, 2A cleavage between P1-2A and 2BC, and maturation cleavage of VP0 to VP2 and VP4, 3C^pro^ can efficiently process all other 10 cleavage sites in FMDV polyprotein although the rate of cleavage varies at different junctions [175]. FMDV 3C^pro^ cleavage sites show great heterogeneity, with cleavage occurring between multiple dipeptides, including Gln-Gly, Glu-Gly, Gln-Leu, and Glu-Ser [176]. By contrast, in other picornaviruses, such as poliovirus, the 3C^pro^ cleavage site located exclusively between Gln-Gly and 3CD^pro^ is implicated as the major viral proteinase in structural protein cleavage [176, 177].

FMDV 3C^pro^ is also associated with inhibition of host cell transcription and translation. As mentioned above, FMDV L^pro^ is involved in eIF4G cleavage, which shuts off host cell gene translation. FMDV 3C^pro^ can also cleave eIF4A, a portion of the cap-binding complex with the function of an RNA helicase [178]. Compared with L^pro^, FMDV 3C^pro^ cleaves eIF4G late in the infection cycle at an alternative site, although it may not be favorable for the translation of viral proteins at this stage [8]. Moreover, 3C^pro^ is the agent that cleaves histone H3 following FMDV infection. Histone H3 is an important component of nucleosome and is crucial in maintaining the conformation of nucleosomes, thereby affecting cellular transcription [179]. FMDV 3C^pro^ also removes 20 N-terminal aa residues from histone H3, resulting in inhibition of host cell transcription [180].

Similar to other picornaviruses, FMDV 3C^pro^ can enter nuclei through its precursor 3CD, which contains a nuclear localization sequence (NLS) in the N-terminus of the 3D^pol^ protein [152]. 3C^pro^ also cleaves multiple factors and regulators. Recent studies found that FMDV 3C^pro^ directly cleaves the 68 kDa Src-associated substrate during mitosis (Sam68), one of the nuclear RNA-binding proteins that participate in viral replication in cells. Immunofluorescent and immunoblot assays revealed that 3C^pro^ removes the NLS-containing C-terminus of the Sam68 protein (~18 kDa). The truncated Sam68 (~50 kDa) was subsequently redistributed into the cytoplasm [181]. Moreover, cytosolic Sam68 directly interacts with the FMDV IRES and enhances the translation of the viral RNA [181].

Other reports associated FMDV 3C^pro^ with innate immune regulation (Fig. 4). Wang et al. provided direct evidence that FMDV 3C^pro^ proteolytically cleaves the nuclear transcription factor kappa B (NF-kB) essential modulator (NEMO), a bridging adaptor protein essential in activating both the NF-kB and IFN-regulatory factor signaling pathways [182], to reduce RIG-I/MDA5 signaling. They found that FMDV 3C^pro^ specifically targets the NEMO Gln_383_ residue, which lies between the C-terminal leucine zipper motif and a zinc finger (ZF) domain [182]. The ZF domain plays a crucial role in fully activating NF-kB and IRFs that orchestrate immune and inflammatory responses [183, 184].

In addition, the picornavirus 3C^pro^ cleaves many other factors and regulators associated with cellular DNA-dependent RNA polymerases I, II, and III, such as TATA-box binding protein, octamer-binding protein, transcription activator p53, cyclic AMP-responsive element binding protein, and DNA polymerase III [7]. Although many of them have not yet been confirmed for FMDV, these data indicated that 3C^pro^ may widely perturb gene transcription and translation in host cells.

### 3D^pol^

FMDV 3D protein, the virus-encoded RNA-dependent RNA polymerase (RdRP) [185], is the catalytic component of RNA replication to synthesize positive- and negative-sense genome and plays an important role in the life cycle of RNA viruses (Fig. 2). 3D^pol^ sequences are highly conserved among the different sero- and subtypes of FMDV [186]. Picornaviruses use a precursor 3CD as a functional intermediate in viral replication, although 3CD contains an active 3C protease component. 3D^pol^ remains inactive until protein processing is complete [187].

Crystal structure analysis revealed that FMDV 3D^pol^ shares similar structure and catalytic mechanism to all other RNA virus-encoded RdRPs of several other families [187]. The overall structure of 3D^pol^ imaginatively resembles a cupped “right hand” consisting of “palm,” “fingers,” and “thumb” subdomains, which determine the correct geometrical arrangement of substrate molecules and metal ions at the active catalytic site [188]. The catalytic site of all RdRPs is contained in the palm domain. This domain is the most highly conserved feature of all known polynucleotide polymerases and is composed of five motifs, a three-stranded antiparallel core β sheet, and flanked by two α helices. This domain is involved in structural integrity, nucleotide recognition and binding, phosphoryl transfer, and priming nucleotide binding. By contrast, the thumb domain consists of the C-terminal region of the polypeptide chain and exhibits the most diverse feature among the known viral RdRPs [187].

Picornavirus replication is initiated in a primer-dependent manner. The protein primer VPg provides the hydroxyl nucleophile and forms a complex with 3D^pol^ or 3CD, termed VPg uridylylation complex, to initiate RNA replication in picornavirus [189, 190]. Biochemical and structural studies revealed that three distinct VPg binding sites on 3D^pol^ are present among different members of this family [191]. In FMDV, VPg binds to the residues in the active site cleft of the polymerase in the uridylylation reaction [190]. Whereas, in coxsackievirus B3 (CVB3), VPg is bound at the base of the thumb sub-domain [192], and in EV71, VPg is found anchored at the bottom of the palm domain of the polymerase [193]. The conformation of RdRPs changes in subtle ways to accomplish three steps cycle of replication elongation process, including nucleotide selection, phosphodiester bond formation and translocation to the next nucleotide for the subsequent round of nucleotide addition (reviewed in [191]).

The structure and biochemical activity of RdRP offer the opportunity to develop very selective anti-drugs against this viral enzyme in FMDV. Reports have indicated that some base and nucleotide analogues, particularly 5-fluorouracil and ribavirin. 5-Fluorouracil, a pyrimidine analogue, is mutagenic for a number of RNA viruses, including FMDV [194]. Ribavirin is also mutagenic for viral RNA polymerases [195] and is useful to eliminate FMDV from persistently infected cells via enhanced mutagenesis [196].

## Conclusions and perspective

FMDV remains a severe pathogen that hampers modern agriculture worldwide, despite the extensive work has been conducted in the past several decades to control it. Traditional vaccine development is limited by the potential risk of virus transmission. Synthetic capsid vaccine, an important novel vaccine against virus infection is limited because of the high potential for genetic and antigenic variation [1, 5]. Specific strategies against FMDV, including antiviral drugs and novel vaccines, are still required in response to repeated epidemic events.

The NSPs and NCEs of FMDV play a critical role in viral proliferation and virus–host interaction. Studies on the involvement of these proteins and elements provided important insights into the molecular mechanism of virus-induced diseases. Nearly all NSPs and NCEs of FMDV are under investigation for their involvement in either virus detection or potential anti-viral strategy development [197–205], although progress is slow. FMDV is unique among the picornavirus family because of its long 5ʹ-UTR, three copies of 3B protein and short 2A protein lacking any protease motifs. Together with our most recent finding about the molecular mechanisms of FMDV entry into host cells [206], these properties possibly contribute to the virulence and pathogenesis of FMDV. In-depth work is still required to further understand the role of these proteins and elements in FMD pathogenesis and virus–host interactions. More extensive research will be helpful to uncover effective antiviral targets.
